# Long-Term Outcome of Covered Stent Implantation for Management of Iliofemoral Vascular Complications in Patients Undergoing Transcatheter Aortic Valve Replacement

**DOI:** 10.1016/j.shj.2025.100489

**Published:** 2025-05-13

**Authors:** Nicholas Johnson, Cara Barnes, Joao Martins, James D. Newton, Adrian P. Banning, Rajesh K. Kharbanda, Dominic P.J. Howard, Ka Hou Christien Li, Sam Dawkins, Thomas J. Cahill

**Affiliations:** a Oxford Heart Centre, Oxford University Hospitals, Oxford, UK; b Department of Vascular Surgery, Oxford University Hospitals, Oxford, UK

**Keywords:** Covered stent, Hemorrhage, TAVR, Vascular access complications

## Abstract

**Background:**

Covered stent implantation has become a common approach for management of iliofemoral vascular complications in patients undergoing transcatheter aortic valve replacement (TAVR), but the long-term outcomes associated with this approach are unknown. The aims of the study were 1) to evaluate the incidence and indication for covered stent placement in patients undergoing TAVR, 2) to assess long-term clinical outcomes after covered stent placement, and 3) to describe the performance of covered stents over long-term follow-up as assessed by Doppler ultrasonography.

**Methods:**

Retrospective cohort study of patients undergoing iliofemoral covered stent implantation at the time of TAVR in a single high-volume UK center.

**Results:**

1277 patients underwent transfemoral TAVR between January 1, 2019, and December 31, 2022. Of these, a total of 54 patients (4.2%) underwent iliofemoral covered stent implantation. Indications for covered stent placement were hemorrhage (39/54, 72.2%), dissection (11/54, 20.4%), and other causes (4/54, 7.4%). Overall, the median follow-up time was 22 ​± ​2.3 months during which no patient required vascular reintervention on the stented limb. Doppler ultrasonography was performed on 27 (71%) of surviving patients at a median of 34 ​± ​2.6 months post-TAVR. There were no cases of stent fracture or complete occlusion. Two patients (7.4%) had evidence of asymptomatic moderate restenosis.

**Conclusion:**

Iliofemoral covered stent placement is a safe and effective means of managing significant vascular complications. Over long-term follow-up, we found no evidence of clinical stent failure requiring reintervention, and a low incidence of subclinical in-stent restenosis.

## Highlights

• Covered stents were highly effective at managing iliofemoral vascular access complications during TAVR.
• Over long-term follow-up after TAVR (median 22 months), there were no cases of clinical stent failure requiring vascular intervention.
• Stent surveillance by duplex ultrasonography confirmed high patency rates of covered stents over long-term follow-up, with no cases of stent fracture or occlusion.

## Graphical abstract

Long-term outcomes of iliofemoral stent implantation for vascular complications in TAVR. Abbreviations: TAVR, transcatheter aortic valve replacement.

**Figure:**
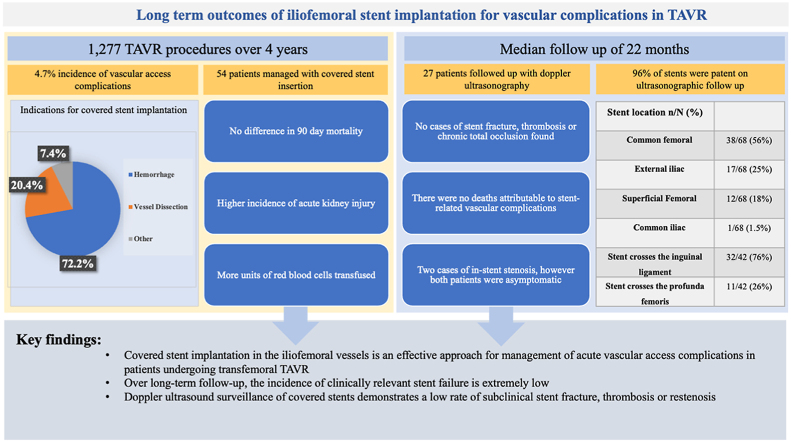

## Introduction

Transcatheter aortic valve replacement (TAVR) has become the treatment strategy of choice for the majority of patients with severe symptomatic aortic stenosis.^1^ Despite progressive improvements in the safety profile of TAVR over the last 2 ​decades, vascular access complications still affect approximately 2% to 5% of patients and are associated with increased morbidity and mortality.^2^, ^3^, ^4^

Injury to the iliofemoral vessels is the most common vascular complication in patients undergoing transfemoral TAVR.^5^ Historically, many vascular complications were managed by open vascular surgical repair, but peripheral vascular complications are increasingly treated in the cardiac catheterization laboratory by covered vascular stent implantation.^6^, ^7^, ^8^ However, there are limited data to support the safety and efficacy of this approach. In addition, the durability of covered stents in the iliofemoral vessels is unclear, particularly when crossing the inguinal ligament, due to the risk of wall stress, stent deformation, and fracture.^9^^,^^10^

The aims of the study were three-fold: first, to evaluate the incidence and indication for covered stent placement in patients undergoing TAVR; second, to assess long-term clinical outcomes after covered stent placement; and third, to describe the performance of covered stents over long-term follow-up as assessed by Doppler ultrasound imaging.

## Methods

### Study Population

The Oxford TAVR registry was used to identify patients who underwent covered stent implantation during transfemoral TAVR between January 1, 2019, and December 31, 2022. Outcome data were retrospectively obtained from the institutional service evaluation database under audit authorization No 5172 from the Oxford University Hospitals National Health Service Trust.

### TAVR Procedure

The choice of transcatheter heart valve was at the operator’s discretion. The devices available during the study period were Lotus Edge (Boston Scientific, Marlborough, Massachusetts, USA), Acurate Neo/Acurate Neo 2 (Boston Scientific, Marlborough, Massachusetts, USA), and the SAPIEN 3/SAPIEN 3 Ultra system (Edwards Lifesciences, Irvine, California, USA). Covered stent choice was at the operator’s discretion. The inguinal ligament was defined by a line between the pubic tubercle to the anterior superior iliac spine of the iliac crest. Initiation and duration of antiplatelet agent use after covered stent implantation was at the operator’s discretion.

### Statistical Analysis

Statistical analyses were performed using SPSS version 29.0.2.0 (IBM Corp. Released 2023. IBM SPSS Statistics for Windows, Version 29.0.2.0, Armonk, New York, USA: IBM Corp). Continuous data were checked for normal distribution using the Shapiro-Wilk test and normal Q-Q plots, then analyzed using either the independent-samples *t*-test if normally distributed or the Mann-Whitney U test if not. Categorical variables were analyzed using the Pearson chi-square test. Significance was set at 95% and *p* values < 0.05 were accepted as being statistically significant.

## Results

**Figure 1:**
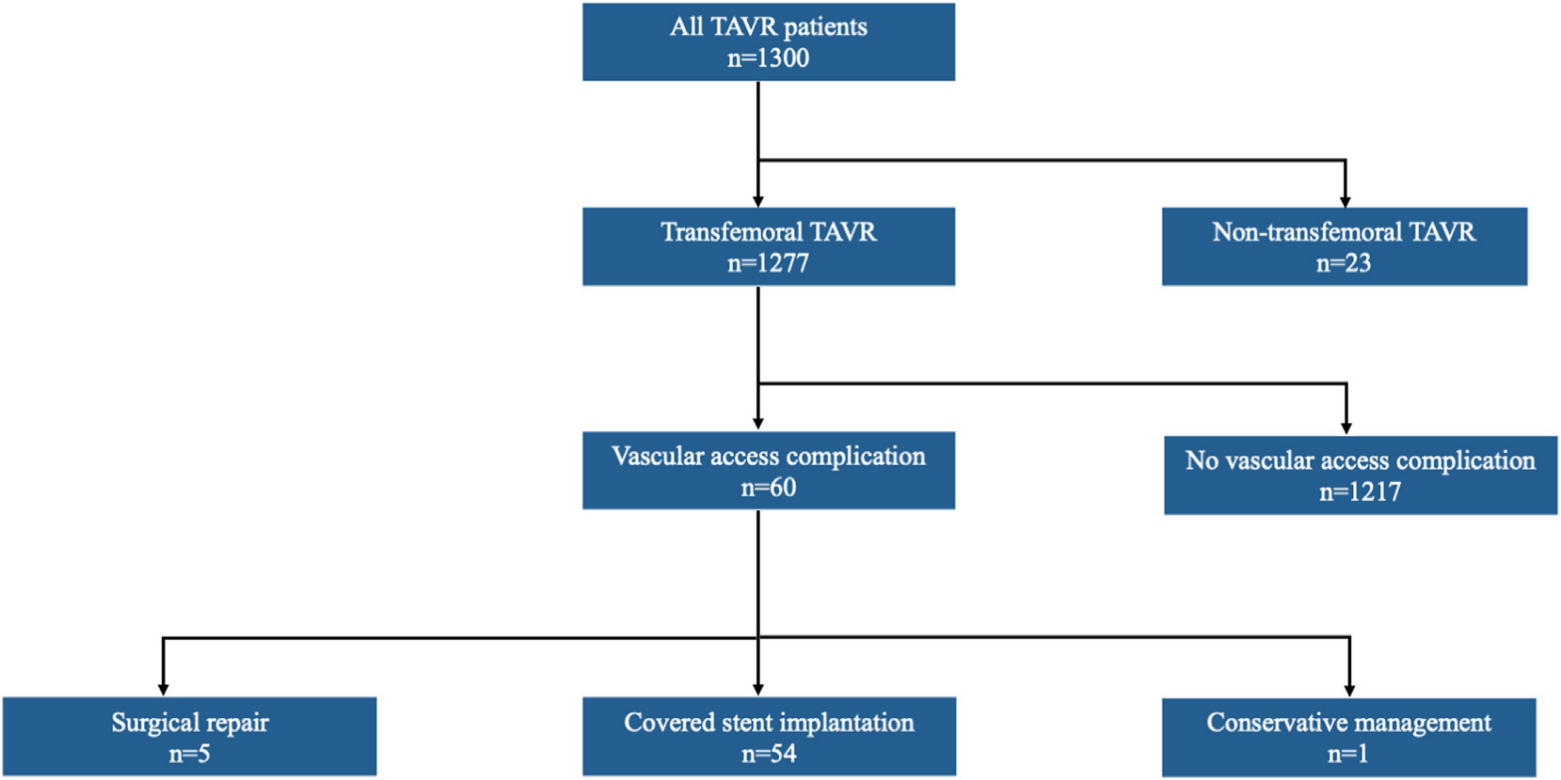
Study flow diagram. Abbreviations: TAVR, transcatheter aortic valve replacement.

A total of 1277 patients underwent transfemoral TAVR between January 1, 2019, and December 31, 2022 (Figure 1). The mean patient age across the cohort was 80.7 ​± ​7.1 years, and 41% were female. A total of 60/1277 patients (4.7%) had a vascular access complication, of which 13 (22%) were classified as Valve Academic Research Consortium 3 major and 47 (78%) were Valve Academic Research Consortium 3 minor. Of patients with a vascular access complication, 54/60 (90%) underwent implantation of a covered vascular stent. Compared to the remainder of the cohort, patients undergoing covered stent implantation did not differ with respect to age, sex, or comorbidities (Table 1). Procedural details were also similar with respect to the transcatheter valve type implanted, valve size, and procedural approach (Table 2).

**Table 1:** Patient characteristics

	Covered stent placed (N ​= ​54)	No covered stent (N ​= ​1223)	*p* value
Age, y	79 ​± ​8	81 ​± ​7	0.33
Sex, female	27/54 (50)	444/1091 (41)	0.18
Prior CABG	6/54 (11)	129/1055 (12)	0.81
Prior myocardial infarction	4/54 (7.4)	82/1063 (7.7)	0.93
Prior PCI	12/54 (22)	187/1058 (18)	0.40
Prior stroke/TIA	6/54 (11)	118/1060 (11)	1.00
Peripheral artery disease	23/54 (43)	379/1059 (36)	0.09
Diabetes mellitus	16/54 (30)	274/1072 (26)	0.51
Current smoker	1/41 (2.4)	17/1017 (1.7)	1.00
COPD	12/54 (22)	264/1061 (25)	0.66
Creatinine, mmol/L	113 ​± ​96	102 ​± ​61	0.74
Prior renal dialysis	2/54 (3.7)	14/1057 (1.3)	0.40
Atrial fibrillation	20/54 (37)	322/1058 (30)	0.31
Aortic valve area (cm^2^)	0.69 ​± ​0.17	0.71 ​± ​0.24	0.78
Left ventricular ejection fraction, %			0.31
Preserved (EF ​≥50%)	38/52 (73)	701/1045 (67)	
Moderately impaired (EF 30%-49%)	12/52 (23)	236/1045 (23)	
Severely impaired (EF <30%)	2/52 (3.8)	108/1045 (10)	

Values presented as mean ± SD or n/N (%).

Abbreviations: CABG, coronary artery bypass grafting; COPD, chronic obstructive pulmonary disease; EF, ​ejection fraction; PCI, percutaneous coronary intervention; TIA, ​transient ischemic attack.

**Table 2:** Stent size, design, and implant location

Stent size	
Mean stent length (mm)	65 ​± ​16
Mean stent diameter (mm)	9 ​± ​2
Stent design/manufacturer	
Self-expanding	
Fluency Plus Endovascular stent graft (BD Interventional, New Jersey, USA)	29/49 (59)
Covera Vascular Covered Stent (BD Interventional, New Jersey, USA)	7/49 (14)
Gore Viabahn Endoprosthesis (Gore & Associates, Delaware, USA)	7/49 (14)
Balloon expandable	
PK Papyrus (Biotronik, Berlin, Germany)	2/49 (4.1)
Bard LifeStream Vascular Covered Stent (Bard Peripheral Vascular Inc, Co, Wexford, Ireland)	2/49 (4.1)
Gore Viabahn VBX Balloon Expandable Endoprosthesis (Gore & Associates, Delaware, USA)	1/49 (2.0)
Atrium Advanta (Getinge, Gothenburg, Sweden)	1/49 (2.0)
Stent location	
Common femoral artery	38/68 (56)
External iliac artery	17/68 (25)
Superficial femoral artery	12/68 (18)
Common iliac artery	1/68 (1.5)
Stent crosses the inguinal ligament	32/42 (76)
Stent crosses the profunda femoris origin	11/42 (26)

Values presented as n/N (%) or mean ​± ​SD.

### Covered Stent Implantation

**Figure 2:**
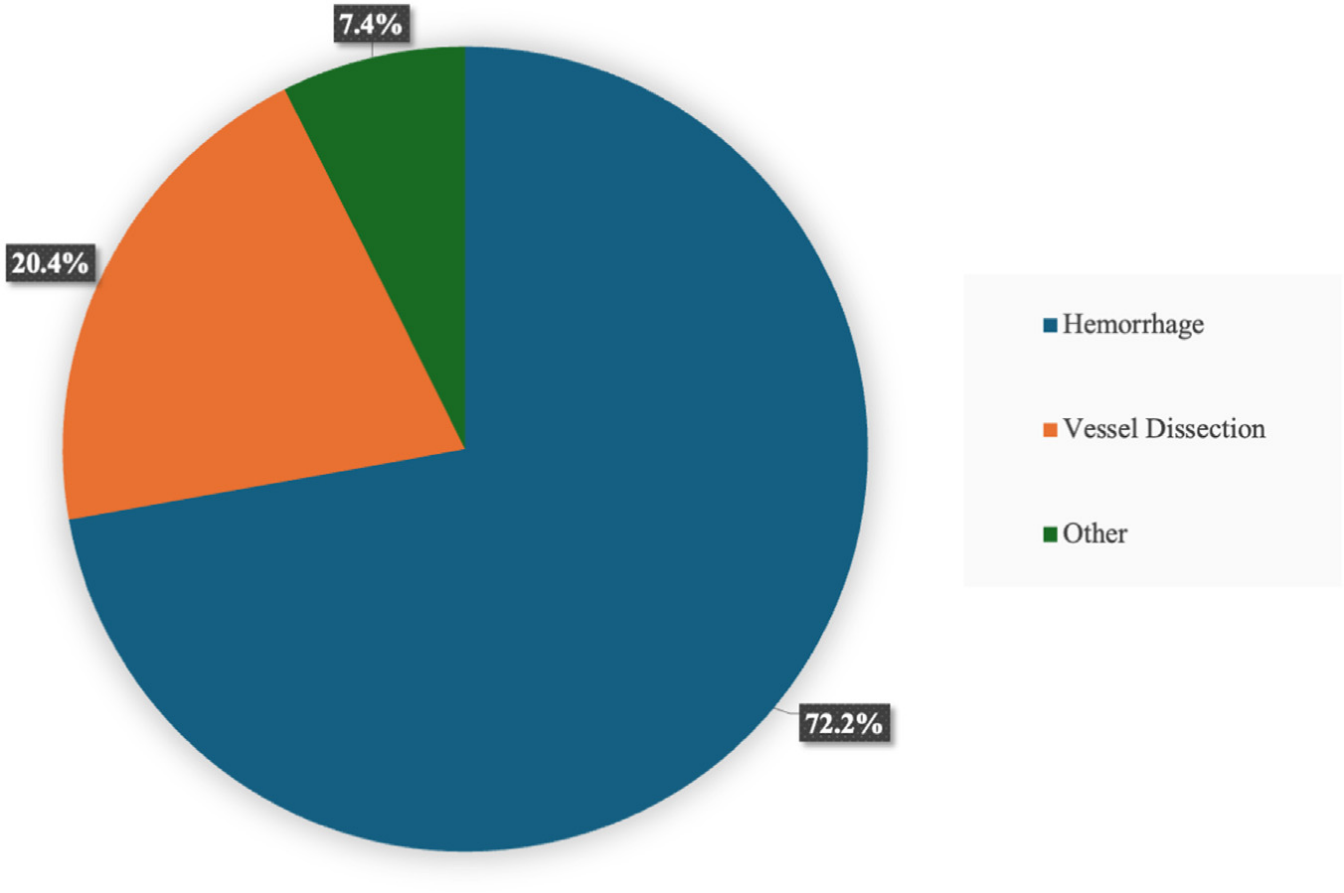
Indications for covered stent implantation at the time of TAVR. Abbreviations: TAVR, transcatheter aortic valve replacement.

The primary indication for iliofemoral covered stent implantation is shown in Figure 2. This was hemorrhage in 39 (72%), dissection in 11 (20%), and acute vessel occlusion in 2 (3.7%) patients, respectively. A total of 60 covered stents were implanted, with 6 patients (11%) having 2 stents inserted (Table 2). The most common site for stent implantation was the common femoral artery (CFA), in 38 patients (70%). The stent type was known for 49 (82%) of stents. Of these, 43 (88%) stents were self-expanding, and 6 (12%) were balloon expandable. The mean stent length was 70 ​± ​24 mm, with a mean stent diameter of 9 ± 2 mm. Thirty-two (59%) patients had stent placement across the inguinal ligament, while 11 (20%) had stent implantation across the profunda femoris artery.

### Short-Term Outcome

Clinical outcomes at 30 days after TAVR by requirement for covered stent placement are shown in Table 3. In the stented cohort, one patient died from progressive shock and multiorgan dysfunction. The incidence of acute kidney injury was higher in the stented group (2/54 [3.9%] vs. 6/1137 [0.5%]; odds ratio [OR]: 7.02; 95% CI: 1.38-35.6; *p* ​= ​0.02), as was the mean number of red blood cell units transfused prior to discharge (0.44 ​± ​0.98 vs. 0.02 ​± ​0.27; *p* ​<​ 0.01). In the covered stent cohort, 2/54 (3.7%) patients were readmitted to the hospital postdischarge due to hematoma formation, with one requiring surgical evacuation and arterial repair. There was no significant difference in in-patient mortality for the stented vs. nonstented groups (1.8 vs. 0.87%; OR: 2.16; 95% CI: 0.27-17.19; *p* ​= ​0.46). There was also no statistically significant difference in 90-day mortality between groups (5.5 vs. 3.5; OR: 1.63; 95% CI: 0.49-5.46; *p* ​= ​0.42).

**Table 3:** In-hospital outcomes

	Covered stent (N ​= ​54)	No covered stent (N ​= ​1223)	*p* value
Death	1/54 (1.9)	10/1153 (0.87)	0.99
Stroke/TIA	1/53 (1.8)	11/1155 (1.0)	1.00
Periprocedural MI∗	0/53 (0)	3/1154 (0.3)	1.00
AKI within 7 d of TAVR	2/51 (3.9)	6/1137 (0.5)	0.049
AKI stage 1	1/51 (2.0)	2/1137 (0.2)	
AKI stage 2	0/51 (0)	1/1137 (0.1)	
AKI stage 3	1/51 (2.0)	3/1137 (0.2)	
New dialysis post-TAVR	1/53 (1.9)	1/1153 (0.1)	0.15
Units of blood transfused	0.44 ​± ​0.98	0.02 ​± ​0.27	<0.01
New PPM postprocedure	2/53 (3.8)	48/1157 (4.1)	1.00

Values presented as mean ± SD or n/N (%).

Abbreviations: AKI, ​acute kidney injury; MI, ​myocardial infarction; PPM, ​permanent pacemaker; TIA, ​transient ischemic attack; TAVR, transcatheter aortic valve replacement.

∗ MI ​≤ ​72 ​h after TAVR.

### Long-Term Outcomes

The median follow-up time was 22 ​± ​16 months (interquartile range: 12.0 to 40.3 months). Over the duration of follow-up, no patient required further vascular intervention on the stented limb. There were no deaths attributable to stent-related vascular complications. One patient re-presented with distal leg ulcers in the stented limb. Doppler ultrasonography demonstrated a 7 cm distal superficial femoral artery occlusion with well-developed collateral vessels, likely due to a combination of diabetes and distal arterial disease. In addition, a proximal profunda femoris artery occlusion was present. The CFA stent was still patent, although with monophasic flow only.

In the overall cohort of patients with vascular access complications (n = 60), 5 patients underwent up-front surgical repair. All 5 patients (100%) survived the vascular complication and were successfully discharged. Over long-term follow-up, 2/5 (40%) required further intervention (in both cases, angioplasty) to the site of vascular repair for symptoms of claudication.

### Doppler Ultrasonography Surveillance

Twenty-seven patients (50%) underwent surveillance Doppler ultrasonography of the stented segment as part of long-term follow-up. The median time to ultrasonographic follow-up was 34 ​± ​12 months (interquartile range: 20.5 to 44.5 months). Doppler ultrasonography demonstrated an overall patency rate of 100%, with all stents demonstrating at least monophasic flow throughout. Two stented patients had a discrete in-stent stenosis of 50%-74% proximally; one stented patient had a distal >50% stenosis; however, no patient was symptomatic. 4 patients (15%) had proximal occlusions of the profunda femoris artery, of which three had been stented across; none of these patients were symptomatic. There were no cases of stent fracture, thrombosis, or chronic total occlusion. The mean Doppler ultrasonography velocities of the stented segments are shown in Table 4.

**Table 4:** Doppler ultrasound findings

Doppler ultrasound index	
Mean common femoral artery in-stent velocity (cm/s)	101.7 ​± ​40
Mean superficial femoral artery in-stent velocity (cm/s)	137.6 ​± ​52
Mean overall in-stent velocity (cm/s)	103.9 ​± ​44
Mean stented limb ABPI	1.12 ​± ​0.19
Mean nonstented limb ABPI	1.04 ​± ​0.32

Values presented as mean ± SD.

Abbreviation: ABPI, ankle brachial pressure index.

## Discussion

The key findings from this study are (1) covered stent implantation in the iliofemoral vessels is an effective approach for management of acute vascular access complications in patients undergoing transfemoral TAVR, (2) over long-term follow-up, the incidence of clinically relevant stent failure is low, and (3) Doppler ultrasound surveillance of covered stents demonstrates a low rate of subclinical stent fracture, thrombosis, or restenosis.

### Prior Investigations of Covered Stent Placement for Patients Undergoing TAVR

Over time there has been a reduction in the incidence of vascular complications after TAVR and a trend toward endovascular management.^11^ Several studies have previously investigated outcomes of covered stent placement. Maurina et al.^12^ reported outcomes of 78 patients who underwent iliofemoral stenting for vascular access complications after TAVR. Over a median follow-up time of 429 days, no cases of percutaneous or surgical new intervention were reported. Similarly, a study of 136 patients undergoing implantation of covered stent grafts across the inguinal ligament in emergent settings demonstrated 99% patency at long-term follow-up.^13^ Furthermore, a combined experience in 71 patients with covered stents after TAVR from Bonn/Copenhagen undergoing duplex sonography at a median of 3.9 years demonstrated a stent patency rate of 100%.^14^ In patients with vascular complications of large bore access, percutaneous treatment compared to surgical repair has demonstrated a high rate of efficacy and safety, with fewer blood transfusions and a faster time to discharge.^15^ There was no difference in the rate of new onset of walking pain, rest pain, and ischemic ulcers during follow-up.

### Current Study–Implications

In this study, covered stent placement was effective at managing iliofemoral hemorrhage and/or dissection in all but one patient, who ultimately died from downstream consequences of multiorgan failure. Over long-term follow-up, one patient developed symptomatic peripheral vascular disease distal to the stented segment, which was not occluded. Although stent placement across the inguinal ligament is considered a relative contraindication, in this cohort of patients, we did not identify a signal for symptoms or subclinical stent failure associated with this approach. This may reflect the reduced mobility in this elderly group of patients compared to a younger, more active group where higher stress force is placed on the stent. Furthermore, stent placement across the profunda femoris origin, with jailing or occluding the profunda branch, remains highly controversial.^16^ In this series, 11 patients underwent emergent covered stent placement across the profunda femoris origin, without clinical sequelae, but in the event of subsequent stent occlusion, there would be extensive limb ischemia extending into the pelvis. For this reason, covered stent placement across the profunda origin should only be considered where there is no option to land proximal to the CFA bifurcation.

There are limited data to guide practice regarding stent design and pharmacology for patients undergoing covered stent implantation in the context of a vascular access complication. In general, self-expanding stents are of high elasticity and flexibility but low radial outward force. Balloon-expandable stents are rigid, support high radial outward force, and are less likely to recoil after postdilatation. In patients with peripheral arterial disease undergoing stent implantation in the common or external iliac artery, the multicenter Iliac, Common, and External Artery Stent Trial demonstrated that self-expanding stents were associated with a reduced risk of 12-month restenosis and target lesion revascularization at 1-year follow-up, compared with balloon-expandable stents.^17^ This trial enrolled a younger population (with a mean age in the mid-60s) compared to the contemporary TAVR population, however, and the applicability of these findings is unclear. Previous studies of covered stent placement in the iliofemoral vasculature for bleeding after endovascular procedures describe good medium-term results with both self-expanding and balloon-expandable covered stent platforms.^14^^,^^18^^,^^19^ On balance, there is currently insufficient evidence to recommend a particular stent design (i.e., either balloon-expandable or self-expanding covered stents) in the iliofemoral vessels in the TAVR population, and the choice largely is governed by operator preference. With respect to pharmacology after covered stent placement, as the TAVR population is frequently high bleeding risk, our routine practice is to treat patients with a single antiplatelet agent (either aspirin 75 mg or clopidogrel 75 mg daily) to continue lifelong. If the patient has an indication for anticoagulation (e.g., atrial fibrillation), we treat with an anticoagulant alone, without an additional antiplatelet.

The optimal strategy for follow-up of patients who have undergone covered stent implantation in the iliofemoral vessels is debated. Our current practice is derived from the American Heart Association/American College of Cardiology guidelines for patients undergoing endovascular procedures for peripheral arterial disease (updated in 2024).^20^ The American College of Cardiology/American Heart Association guideline provides a class IIa recommendation for routine ankle brachial index and arterial duplex ultrasound surveillance after stent implantation, at 1-3 months, 6 months, 12 months, and then annually, regardless of whether the patient has further lower limb symptoms. As many of the patients undergoing TAVR are elderly and live a substantial distance from our center, we recommend a pragmatic approach of annual clinical review with duplex ultrasonography and ankle-brachial index. If the patient has lower limb symptoms, we expedite investigation as appropriate.

### Limitations

This was a single-center, observational cohort study with limited patient numbers. Some demographic and patient factors of potential interest, such as body mass index, were not available within the data set. Follow-up Doppler ultrasonography was not available for all patients. There was heterogeneity in the stent design and the use of antiplatelet/anticoagulant agents. Computed tomography follow-up may be more sensitive than ultrasonography for detection of stent fracture. Very long-term follow-up of larger patient cohorts is required before this approach can be expanded to younger patients undergoing TAVR. Lastly, the findings may not be applicable to younger patients, who make up an increasing proportion of patients undergoing TAVR in some parts of the world.

## Conclusions

Iliofemoral stent placement is a safe and effective strategy for management of vascular access complications after the TAVR procedure, with low rates of stent failure over long-term follow-up.

## Ethics Statement

All patients were managed in line with established local guidelines. Data were retrosectively obtained from the institutional prospective service databases under audit authorisation No 5172 from the Oxford Univeristy Hospitals NHS foundation trust.

## Funding

The authors have no funding to report.

## Disclosure Statement

N. Johnson reports a relationship with Oxford Heart Centre, Oxford University Hospitals, Oxford, UK, that includes: employment. C. Barnes reports a relationship with Oxford Heart Centre, Oxford University Hospitals, Oxford, UK, that includes: employment. J. Martins reports a relationship with Oxford Heart Centre, Oxford University Hospitals, Oxford, UK, that includes: employment. J.D. Newton reports a relationship with Oxford Heart Centre, Oxford University Hospitals, Oxford, UK, that includes: employment. A.P. Banning reports a relationship with Oxford Heart Centre, Oxford University Hospitals, Oxford, UK, that includes: employment. R.K. Kharbanda reports a relationship with Oxford Heart Centre, Oxford University Hospitals, Oxford, UK, that includes: employment. D.PJ. Howard reports a relationship with the Department of Vascular Surgery, Oxford University Hospitals, Oxford, UK that includes: employment. K.H.Christien Li reports a relationship with Oxford Heart Centre, Oxford University Hospitals, Oxford, UK, that includes: employment. S. Dawkins reports a relationship with Oxford Heart Centre, Oxford University Hospitals, Oxford, UK, that includes: employment. T.J. Cahill reports a relationship with Oxford Heart Centre, Oxford University Hospitals, Oxford, UK, that includes: employment.
