# Supplementary material for: Long-Term Outcome of Covered Stent Implantation for Management of Iliofemoral Vascular Complications in Patients Undergoing Transcatheter Aortic Valve Replacement
Source: Struct Heart. 2025 May 13;9(9):100489. doi: 10.1016/j.shj.2025.100489 (PMC12391694; doi:10.1016/j.shj.2025.100489)
Supplement: Supplementary Table 1 [file mmc1.docx]

**Supplementary Tables**

|  | **Covered stent placed**  **(N = 54)** | **No covered stent placed (N (N = 1223)** | **p-value** |
| --- | --- | --- | --- |
| **Valve Type** |  |  | 0.42 |
| **Boston Scientific Acurate Neo** | 14/53 (26) | 199/1166 (17) |  |
| **Edwards Sapien 3** | 33/53 (62) | 848/1166 (73) |  |
| **Boston Scientific Lotus** | 5/53 (9.4) | 108/1166 (9.2) |  |
| **Edwards Sapien XT** | 0/53 (0) | 6/1166 (0.5) |  |
| **Abbott Portico** | 0/53 (0) | 6/1166 (0.5) |  |
| **Valve Size, mm** | 25 ± 2 | 26 ± 2 | 0.25 |
| **23mm** | 19/53 (36) | 353/1192 (30) |  |
| **25mm** | 6/53 (11) | 105/1192 (8.8) |  |
| **26mm** | 14/53 (26) | 248/1192 (21) |  |
| **27mm** | 4/53 (7.5) | 118/1192 (9.9) |  |
| **29mm** | 10/53 (19) | 258/1192 (22) |  |

**Supplementary Table 1: TAVR valve prosthesis**
